# Supplementary material for: Controlling supercurrents and their spatial distribution in ferromagnets
Source: Nat Commun. 2017 Dec 12;8:2056. doi: 10.1038/s41467-017-02236-2 (PMC5727026; doi:10.1038/s41467-017-02236-2)
Supplement: Supplementary file 1 — Supplementary Information [file 41467_2017_2236_MOESM1_ESM.pdf]

Supplementary Figures

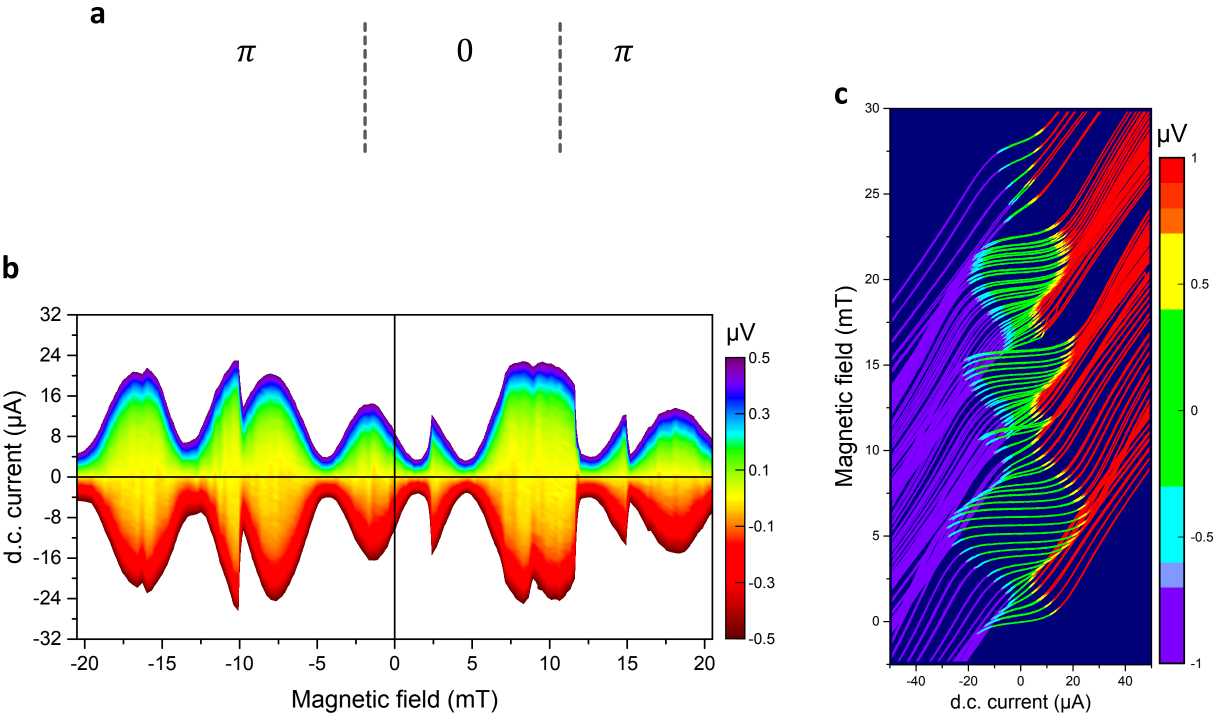

Supplementary Figure 1 | Interference patterns from the virgin magnetic state. (**a**) Disordered magnetic state of Ni before conditioning (schematic). The stochastic magnetic orientation of Ni on each side of the trench can lead to the formation of multiple 0 and π segments across the junction. (**b**) Supercurrent interference pattern of the virgin magnetic state, measured while sweeping the out-of-plane field from negative to positive $30\ \mathrm{mT}$ in steps of $0.3\ \mathrm{mT}$. On average, the supercurrent is suppressed for small fields (below $5\ \mathrm{mT}$) in both field directions. The interference pattern is characterized by random discontinuities. These irregularities are shown more clearly in (**c**) which shows individual $I-V$ curves taken while scanning the field from $28\ \mathrm{mT}$ back to zero. The curves are given an offset to represent the field they were measured at. All measurements are taken at $T = 2.1\ \mathrm{K}$.

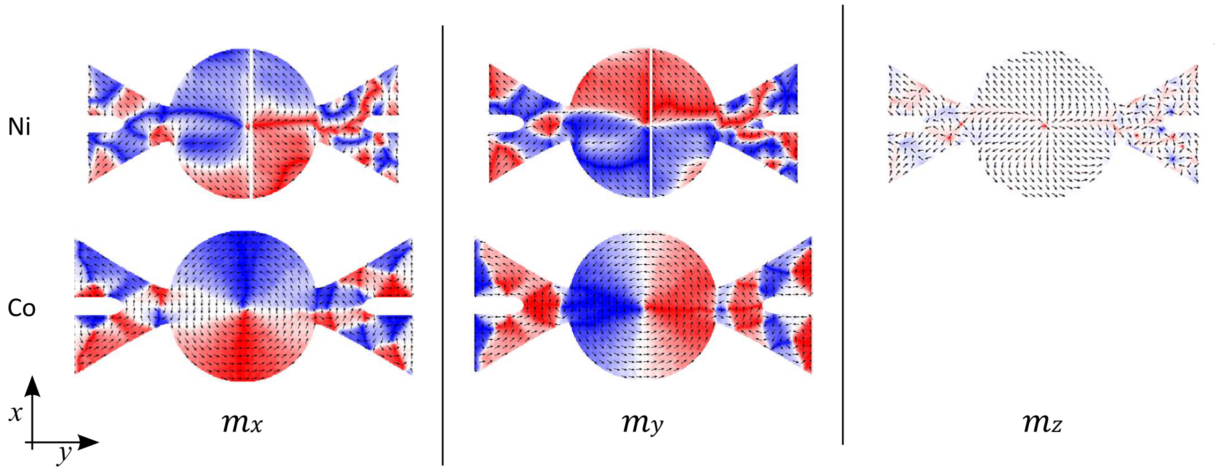

Supplementary Figure 2 | Micromagnetic simulations with an in-plane field. Top views of the magnetic states of Co and Ni layers obtained from OOMMF simulations. The leads are included in the design to produce a more realistic shape anisotropy, needed to accurately describe the system under an in-plane field. Individual components of the magnetization vector $\mathbf{m}$ are plotted separately for clarity. The pixel colour scheme, red-white-blue, scales with the magnitude of each component. The red and blue pixels represent positive and negative values respectively. Out of plane magnetization (m_z) is generally suppressed, except at the vortex core where both layers have a highly localized out of plane component. In the actual device, the trench that forms the junction is slightly off-centred. This feature is accounted for in the simulations by placing the gap in Ni 40 nm away from the centre of the disk.

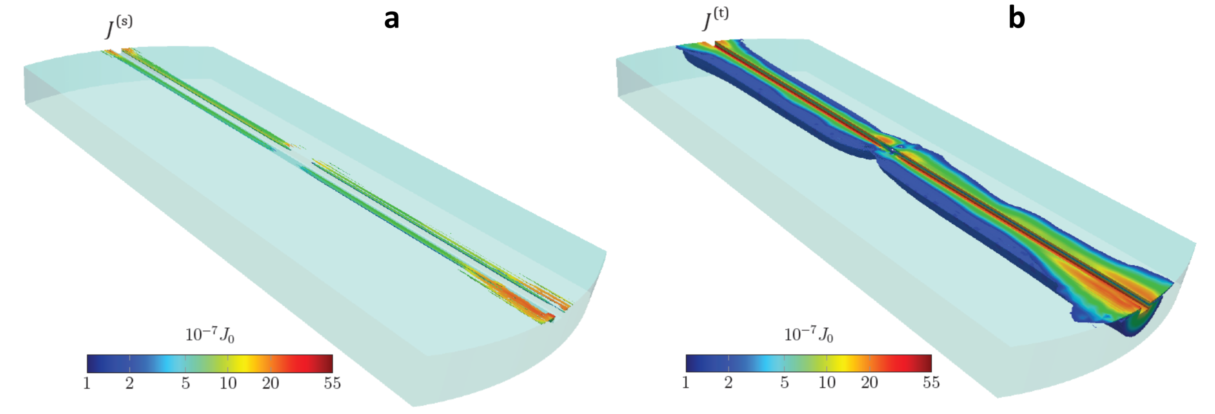

Supplementary Figure 3 | Simulated contributions of singlet and triplet supercurrents (**a**) Magnitude of the current density generated by singlet Cooper pairs, $J^{(s)} = \mathbf{J}^{(s)}$, which is greatly suppressed except for in the immediate vicinity of the superconductors. (**b**) Magnitude of the current density generated by triplet Cooper pairs, $J^{(t)} = \mathbf{J}^{(t)}$. For clarity, currents lower than $10^{-7}J_0$ have been removed, which explains why no singlet current is observed in the trench. It is noted that while the total current $\mathbf{J} = \mathbf{J}^{(s)} + \mathbf{J}^{(t)}$ is conserved, $\mathbf{J}^{(s)}$ and $\mathbf{J}^{(t)}$ are generally not. This is due to the magnetization, which causes oscillations between the singlet and triplet states.

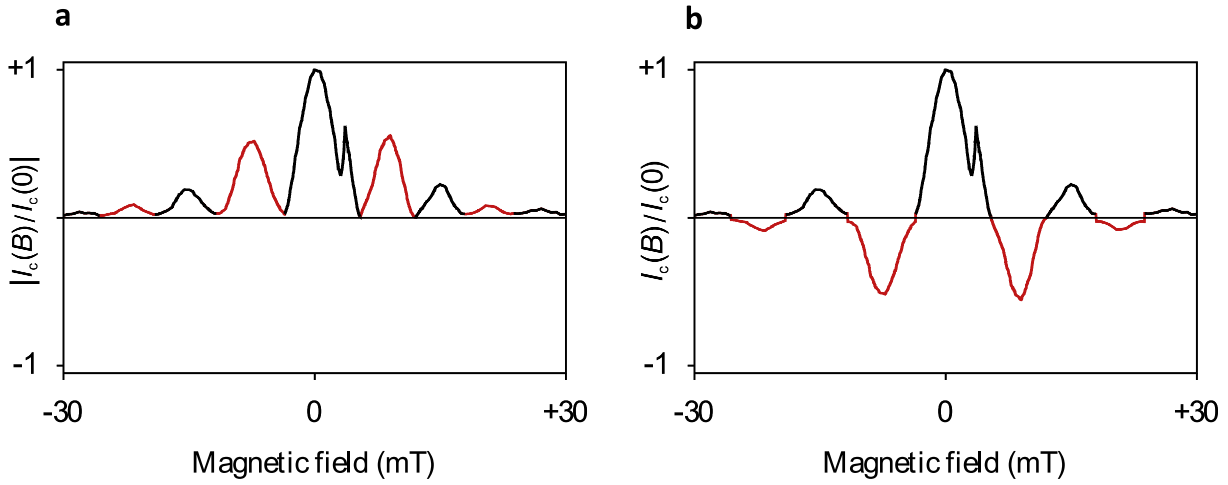

Supplementary Figure 4 | Recovering the complex critical current. (**a**) The (unsigned) $|I\ (B)|$ pattern extracted from $I - V$ measurements. (**b**) The signed $I_c(B)$ interference pattern reconstructed by flipping the signs of alternate lobes as in ref. 1. The data were taken at 2.1 K.

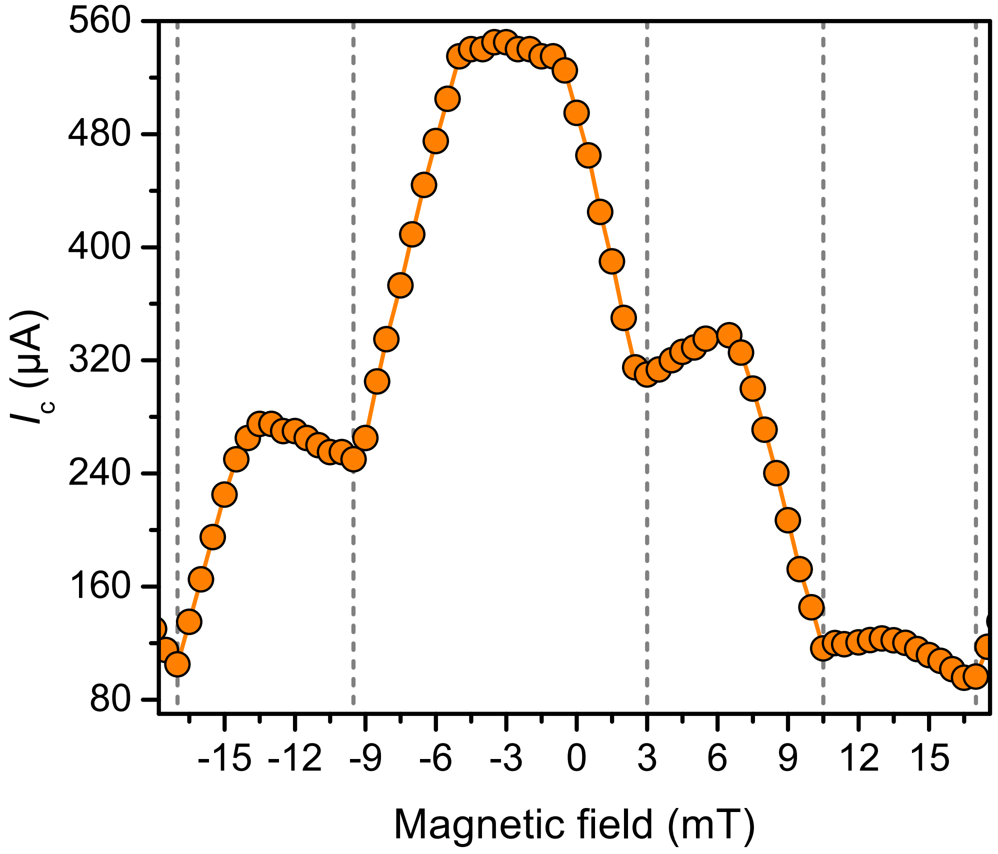

Supplementary Figure 5 | Control experiment. Supercurrent interference pattern measured at $T = 2.1$ K from a control device that was processed in parallel with the one presented in the main text. Deposited together on one substrate, the same multilayer of Pt(7 nm)/Nb(45 nm)/Ni(1.5 nm)/Cu(5 nm)/Co(60 nm) was used in both devices. The Ga^+ dose applied in milling the weak link for the control device was reduced by 50%. This provides a nonmagnetic pathway in the weak link, where singlet current is not suppressed, and can therefore dominate the transport. The result is a junction with a substantially higher critical current, showing single-slit interference pattern.

Supplementary Notes

Supplementary Note 1 | Numerical simulations of the critical current.

To calculate the critical current we use the quasiclassical approximation in the diffusive limit, which yields the Usadel equation[5]

$$D\,\nabla\,\hat{\boldsymbol{g}}\,\nabla\,\hat{\boldsymbol{g}} + i[\varepsilon\,\hat{\boldsymbol{\rho}}_3 + \hat{\boldsymbol{\sigma}}\cdot\boldsymbol{h}\,,\hat{\boldsymbol{g}}] = 0 \qquad (1)$$

where D is the diffusion constant and ε is the quasiparticle energy. The magnetization texture from the micromagnetic simulations are represented as an exchange field $\mathbf{h} = \mathbf{h}(\mathbf{r})$. Furthermore we have defined $\hat{\boldsymbol{\sigma}} = \mathrm{diag}(\boldsymbol{\sigma}\,,\boldsymbol{\sigma}^*)$, where $\boldsymbol{\sigma}$ is a vector of Pauli matrices, and $\hat{\boldsymbol{\rho}}_3 = \mathrm{diag}(1,1,-1,-1)$. From $\hat{\mathbf{g}} = \hat{\mathbf{g}}\,(\mathbf{r},\varepsilon)$, the 4×4 retarded Green function matrix in Nambu $\otimes$ spin space, the equilibrium current density may be computed as

$$\mathbf{J} = \frac{N_0 e D}{2}\int d\varepsilon\ \mathrm{Re}\ \mathrm{Tr}\{\hat{\boldsymbol{\rho}}_3\ \hat{\mathbf{g}}\ \nabla\,\hat{\mathbf{g}}\}\tanh\left[\frac{\beta\varepsilon}{2}\right] \qquad (2)$$

where N_0 is the density of states at the Fermi level, and $\beta = 1/k_B T$. We neglect the inverse proximity effect, and assume that the superconductors on each side of the trench are large enough to be approximated as bulk. In the calculations, we have used that the critical current is approximately found for a phase difference between the superconductors of $\Delta\phi = \frac{\pi}{2}$. For simplicity, we use transparent boundary conditions between the Ni and the Co layer, whereas we use the low-transparency Kupriyanov-Lukichev boundary conditions[6] at the Ni-Nb interface.

In the modeling of the geometry, we have assumed an effective superconducting coherence length of $\xi = 10\,\mathrm{nm}$, so that the radius of the circular disk becomes $R = 50\xi$. In the direction crossing the trench, the model has been truncated to a width of $W = 40\xi$ to reduce the model size. This has been done under the assumption that

any contribution to the current from the removed regions is negligible due to the vast distance to the opposite superconductor. The thickness of the Ni and the Co layers have been set to ξ and 6ξ, respectively, and the width of the trench is 2ξ. The Ni thickness is set larger than in the actual experiment to avoid unnecessarily small elements in the Ni-region, which would substantially increase the calculation time. Although this yields lower values for the triplet current, the purpose of our calculation here is to identify the origin of this current; and not its absolute magnitude.

The spatial distribution of the magnetization in both the Ni and the Co layer are accurately mapped onto the 3D mesh via the exchange field $\mathbf{h}$, where an amplitude of $|\mathbf{h}| = 30\,\Delta \simeq 46\,\mathrm{meV}$ was used. While this is significantly lower than typical exchange fields in Co, it is still sufficient to quench the contribution of singlet Cooper pairs to the current density. To verify this, we make use of the fact that the supercurrent density generated by the singlet $\mathbf{J}^{(\mathrm{s})}$ and triplet $\mathbf{J}^{(\mathrm{t})}$ Cooper pairs contribute additively $\mathbf{J} = \mathbf{J}^{(\mathrm{s})} + \mathbf{J}^{(\mathrm{t})}$. The two components are presented in Supplementary Figure 3. It is clear that the current density of singlet pairs rapidly vanishes away from the superconductors. In contrast, the triplet current density maintains an appreciable value over a substantially larger region, indicating that triplet Cooper pairs are the primary means of transport. The results will therefore be qualitatively the same for a more realistic strength of the exchange field. The advantage of using the reduced value is that the current densities become larger, which in turn make the numerical calculations less resource intensive.

The finite element analysis was carried out using 27-node hexagonal volume elements, and the Green function is interpolated within each element by means of

second order Lagrange polynomials. This means that the current density within each element is interpolated by linear polynomials. To ensure that the spatial distribution of the current density is accurately resolved, we use a refined mesh in a region surrounding the trench, as is shown in Fig. 2a in the main text. For more details regarding the finite element analysis of three-dimensional superconducting heterostructures, please consult ref. 7.

Supplementary Note 2 | Interference patterns from the virgin magnetic state. Prior to conditioning the magnetization, supercurrent interference patterns were measured using small out-of-plane fields. These are presented in Supplementary Figure 1. In contrast to the conditioned sample, $I_c(B_z)$ is generally suppressed around zero field. We observe two maxima, which always occur at fields higher than $5\ \mathrm{mT}$. Note that this offset cannot be attributed to remnant fields from the ferromagnet. The applied field for the interferometry measurements is not sufficient to have an appreciable influence on the magnetization of either layer. This is verified by SQUID magnetometry and ferromagnetic resonance experiments.

It has been proposed that the phase of triplet correlations in a S/F'/F/F'/S junction such as ours, is determined by the relative magnetic orientation of the F and F' layers on each side[2]. On the other hand, this unusual interference pattern, with two maxima and suppressed I_c at zero field, is the characteristic of a junction with multiple parallel 0 and π channels[3,4]. This condition could be fulfilled in the virgin state, where the arbitrary orientation of Ni and Co magnetization can lead to random formation of multiple 0 and π segments across the junction. These interference patterns are also characterized by irregular discontinuities, which could be attributed to the arbitrary arrangement of the 0 and π segments.

Remarkably, we find these features to disappear altogether after conditioning the sample: $I_c(B_z)$ turns into a highly regular and reproducible SQUID pattern, with maximum I_c consistently appearing at $B_z = 0$. This pronounced dependence on magnetic conditioning was absent for junctions where singlet correlations dominated the transport: no significant changes in the interference pattern or the maximum value of I_c were observed.

Supplementary Note 3 | Fourier analysis of supercurrent density profiles.

As shown by Dynes and Fulton[1], the supercurrent density profile $J(x)$ can be determined from the superconducting interference pattern $I_c(B)$ using a Fourier transform:

$$J(x) \sim \int_{-\infty}^{+\infty} dB\, I_c(B)\, e^{\frac{2\pi i L B x}{\Phi_0}} \qquad (3)$$

Here, the coordinate system is defined such that the magnetic field B is applied along the z-axis, the critical current I_c is measured along the y-axis, and the current distribution $J(x)$ can then be determined along the x-axis. The equation also depends on the effective length L of the junction and the flux quantum $\Phi_0 = h/2e$. Note that $I_c(B)$ is the signed critical current, where the sign is determined from the experimentally measured $|I_c(B)|$ by assuming that it consists of alternating positive and negative lobes, as described in more detail in ref. 1. This procedure is justified when the interference pattern consists of well-defined maxima separated by deep minima, as is the case for our measurements.

The original method by Dynes and Fulton was derived for a rectangular junction where the dimensions of each superconductor are much larger than the London penetration depth λ. In that case, the effective junction length $L = 2\lambda + d$, where d is

the thickness of the barrier between the superconducting leads. In our case, however, the junction is cylindrical and the current distribution not uniform, so the length (which determines the amount of flux to be screened) is not well defined. We therefore performed the Fourier analysis without making any assumptions regarding the value of L, but instead assumed that the position along the x-axis where we obtained $J(x) \to 0$ likely corresponded to the junction ends $x \approx \pm R$, where R is the cylinder radius. From this, we obtained an estimate $L \approx 180$ nm for the effective junction length. This value is somewhat lower than expected for a uniform rectangular junction: in that case the effective area is $2RL$ while the first minimum in $I_c(B_z)$ is at 7.8 mT, yielding $L \approx 270$ nm. Both numbers are of correct order of magnitude: the value of λ for a 50 nm Nb film is about 110 nm[8] so $2\lambda + d$ is 240 nm. If we were to take the sharp drop in the current density profile as the sample edge, L would become less than 100 nm, which appears to be too low in view of the value of λ.

The SQI experiments are carried out by measuring the voltage as a function of current for a given applied magnetic field, i.e. $V(I,B)$. The critical current $|I_c(B)|$, used for the Fourier analysis, is obtained by extracting a contour for a small but finite voltage threshold $V(I_c,B) > 0.3\ \mu\mathrm{V}$. Experimentally we find this criterion to be optimal for reducing noise effects that distort the shape of $I_c(B)$. The result is then adjusted to the y-axis so that $|I_c(B)| = 0$ at the nodes between the lobes of the interference pattern. This is to account for the artificial offset introduced by the $0.3\ \mu\mathrm{V}$ threshold voltage. We then recover the complex critical current $I_c(B)$, by switching the sign of every other lobe of the measured $|I_c(B)|$. The original $|I_c(B)|$ and the signed $I_c(B)$ curves are shown side-by-side in Supplementary Figure 4.

Note that the measured $I_c(B)$ may slightly deviate from a perfectly symmetric pattern, and yield a complex supercurrent distribution $J(x)$ after Fourier transformation. This apparent asymmetry however is predominantly caused by experimental noise. We therefore discard the complex phase $J(x)$ to approximate the supercurrent distribution profile by $|J(x)|$, shown in Fig. 3f of the main text.

Supplementary References

1. Dynes, R. C. & Fulton, T. A. Supercurrent density distribution in Josephson junctions. *Phys. Rev. B* **3,** 3015-3023 (1971).

2. Houzet, M. & Buzdin, A. I. Long range triplet Josephson effect through a ferromagnetic trilayer. *Phys. Rev. B* **76,** 060504 (2007).

3. Smilde, H. J. H., Ariando, D. H. A., Blank, G. J., Gerritsma, H., Hilgenkamp, H. & Rogalla, H. *d*-Wave–Induced Josephson current counterflow in $YBa_2Cu_3O_7$/Nb zigzag junctions. *Phys. Rev. Lett.* **88,** 057004 (2002).

4. Gürlich, C. *et al.* Visualizing supercurrents in ferromagnetic Josephson junctions with various arrangements of 0 and π segments. *Phys. Rev. B* **81,** 094502 (2010).

5. Usadel, K. D. Generalized diffusion equation for superconducting alloys. *Phys. Rev. Lett.* **25,** 507–509 (1970).

6. Kuprianov, M. Y. & Lukichev, V. F. Influence of boundary transparency on the critical current of 'dirty' SS'S structures. *Sov. Phys. JETP* **67,** 1163 (1988).

7. Amundsen, M. & Linder, J. General solution of 2D and 3D superconducting quasiclassical systems: coalescing vortices and nanoisland geometries. *Sci. Rep* **6,** 22765; doi: 10.1038/srep22765 (2016).

8. Gubin, A. I., Il'in, K. S., Vitusevich, S. A., Siegel, M. & Klein, N. Dependence of magnetic penetration depth on the thickness of superconducting Nb thin films. *Phys. Rev. B* **72,** 064503 (2005).
